# Comparison of 3-Tier Cytological Grading Systems for Breast Carcinoma

**DOI:** 10.1155/2014/252103

**Published:** 2014-03-12

**Authors:** Dinisha Einstien, B. O. Parijatham Omprakash, Hemalatha Ganapathy, Sadaf Rahman

**Affiliations:** Department of Pathology, Sree Balaji Medical College and Hospital, No. 7, CLC Works Road, Chrompet, Chennai 44, India

## Abstract

*Background*. Fine-needle aspiration cytology plays a major role in the primary diagnosis of breast carcinoma. Cytological grading of the smears can provide valuable prognostic information and aid in planning the management options. *Aim*. To evaluate various 3-tier cytological grading systems and to determine the best possible system which is reliable and objective for use in routine practice. *Materials & Methods*. 72 fine-needle aspiration smears of breast carcinomas were graded by two pathologists and compared with the histologic grading by Nottingham modification of Scarff-Bloom-Richardson method. Concordance and correlation studies were done. Kappa measurement of interobserver agreement was also done. *Results*. Robinson's method showed a better correlation (77.7%) and substantial Kappa value of agreement (*κ* = 0.61) with Bloom Richardson's histological grading method in comparison to the other methods, closely followed by Fisher's method. Fisher's method showed better interobserver agreement (84.7%, *κ* = 0.616) compared to the other systems. *Conclusions*. Robinson's method of cytological grading in fine-needle aspiration smears of breast carcinoma is simpler, multifactorial, and feasible, hence being preferable for routine use according to our study.

## 1. Introduction

In India, breast cancer is the second most common malignancy in females, next to cervical cancer [1]. The study by Khanna et al. has shown increasing incidence in breast cancer, especially in the younger age group [2]. Early diagnosis and prompt therapy are important to increase the survival of the patients. Surgical biopsy specimens serve as the “gold standard” for validating the diagnostic criteria and the value of histological grading has been widely accepted [3]. Fine needle aspiration (FNA) cytology is used for the preoperative diagnosis of breast malignancies and its role in determining the prognosis is being studied by various authors. The National Cancer Institute, Bethesda, sponsored conference had also recommended that the tumour grading on FNA material should be incorporated in FNA reports for prognostication [4]. Black et al. in 1955 introduced the concept of nuclear grading, which was modified and applied in cytological smears by Fisher et al. [5, 6]. Numerous two-tier and three-tier systems have been proposed for the cytological grading of breast tumors, but no single system is currently adapted for use in the routine evaluation of cytological aspirates of breast carcinoma.

In the present study, various three-tier cytological grading systems were studied and compared to arrive at a simple, effective, reliable, and feasible system for the cytological grading of breast carcinoma.

## 2. Materials and Methods 

This retrospective and prospective study was conducted in the Department of Pathology, Sree Balaji Medical College and Hospital, Chrompet, Chennai, during the period of January 2012 to December 2013. A total of 157 FNAC samples of breast tumors were received. Out of which, 72 cases had histopathological correlation and were included in the present study. The alcohol-fixed, Hematoxylin & Eosin stained FNAC smears were studied and graded independently by two pathologists. The following 3-tier grading systems were assessed—Fisher's modification of Black's nuclear grading, Robinson's method, Dabbs and Silverman cytological grading, and the grading systems proposed by Khan et al., Taniguchi et al., Mouriquand and D. Pasquier, and Howell et al.

**Figure 1:**
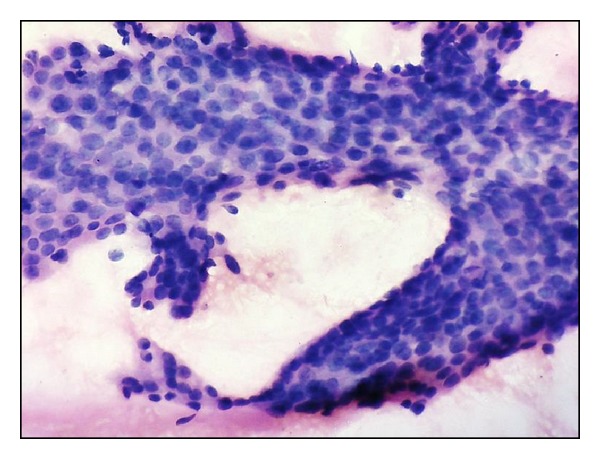
Fisher's grade I, Uniform cells, with fine chromatin and inconspicuous nucleoli (H&E x400).

**Figure 2:**
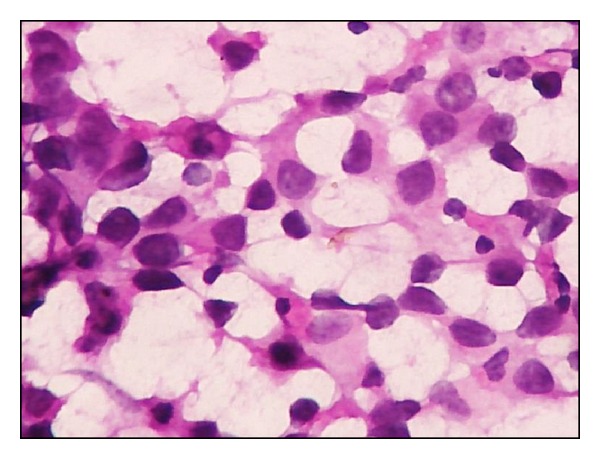
Fisher's grade III. Marked anisonucleosis, large nuclei with clumped chromatin and prominent nucleoli (H&E ×400).

In Fisher's modification of Black's nuclear grading [6], (Figures 1 and 2) five parameters—nuclear shape, chromatin, nucleoli, mitosis, and nuclear size, were graded I to III.

**Figure 3:**
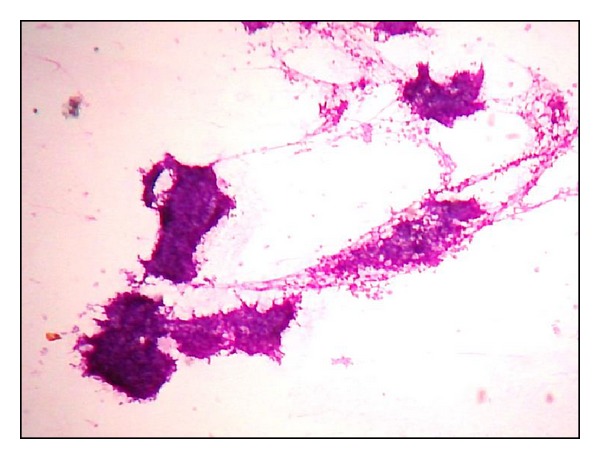
Robinson's grade I, Cells in clusters (H&E ×40).

**Figure 4:**
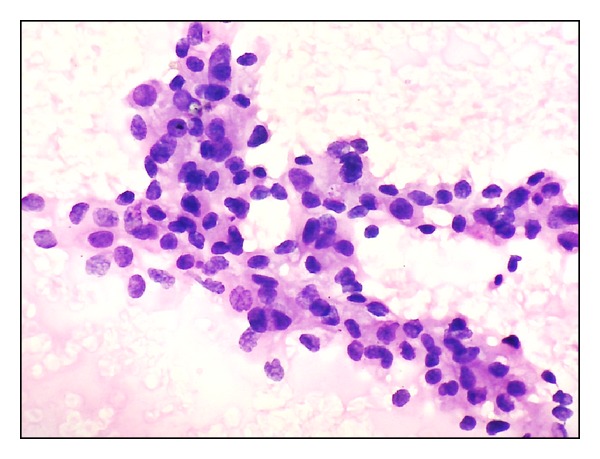
Robinson's grade II. Irregular nuclear margin with folds, granular chromatin (H&E ×400).

**Figure 5:**
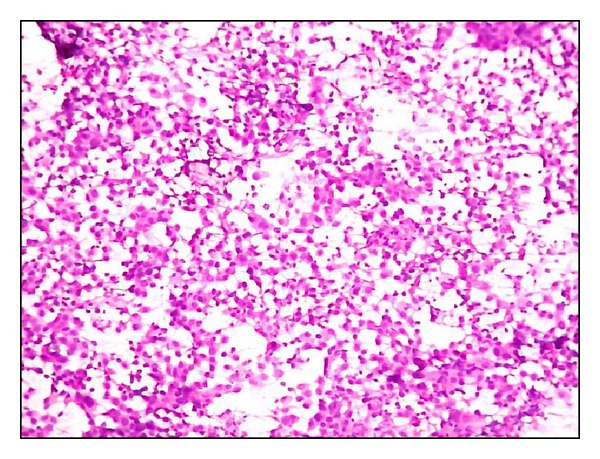
Robinson's grade III. Cells predominantly in singles (H&E ×40).

In Robinson's grading system [7, 8], (Figures 3, 4, and 5) six parameters, namely—cell dissociation, cell size, uniformity, nucleoli, nuclear margin, and chromatin, were given a score of 1–3. A total score in the range of 6–11 was graded as grade I, 12–14 as grade II, and 15–18 as grade-III.

**Figure 6:**
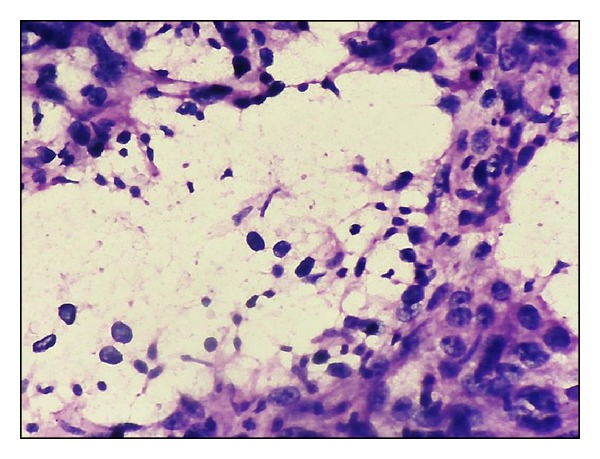
Dabbs and Silverman grade III. Marked pleomorphism, irregular nuclear membrane, and coarse chromatin with clearing (H&E ×400).

**Figure 7:**
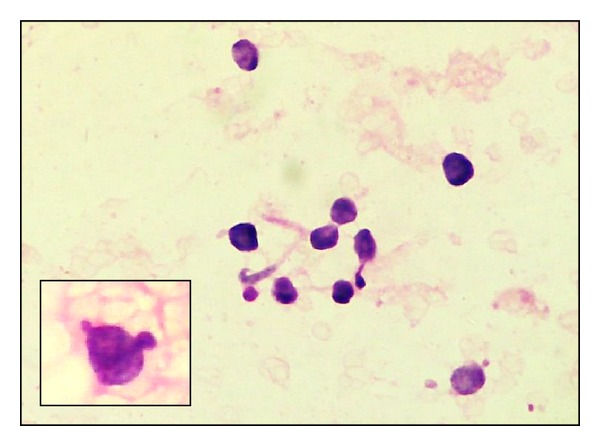
Khan's grading. Naked tumour nuclei (H&E ×400). Inset - nuclear budding from another field of the same smear.

**Figure 8:**
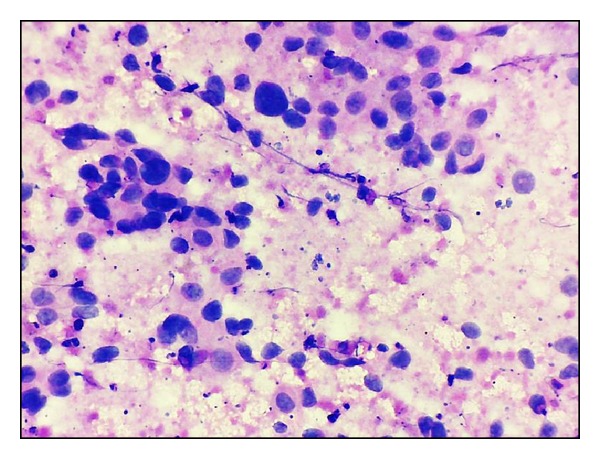
Taniguchi's grade III. Increased N : C ratio, marked pleomorphism, coarse chromatin, and prominent nucleoli with necrosis (H&E ×400).

Dabbs and Silverman method [9] (Figure 6) employed grades from I to III, depending upon the nuclear size and shape, nuclear membrane, chromatin, and nucleoli. Khan et al. [10] proposed a system, which included pleomorphism, nuclear size, nuclear margins, nucleoli, naked tumor nuclei (Figure 7), and mitotic count. The parameters were scored from 1 to 3 and total scores in the range of 6–10, 11–14, and 15–18 were regarded as grade I, grade II, and grade III, respectively whereas Taniguchi et al. [11] included 7 parameters—necrosis (absent - score 0, present - score 1) (Figure 8), cellular size, N : C ratio, nuclear pleomorphism, nucleoli, chromatin granularity, and chromatin density scored from 1 to 3 and total in the range of 6–9, 10-11, and 12–19 as grades I, II, and III, respectively.

**Figure 9:**
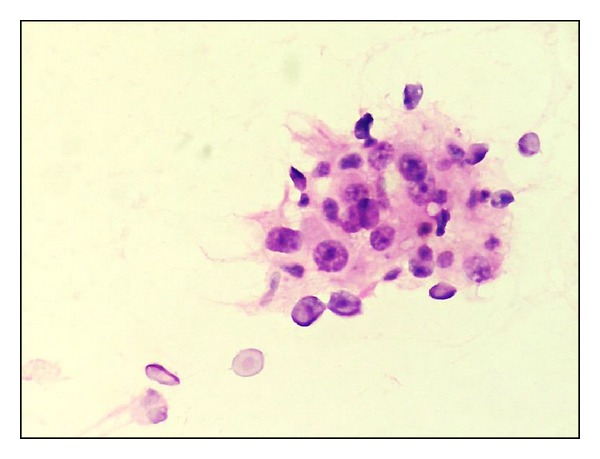
Mouriquand's grade III. Anisonucleosis, enlarged nucleoli (H&E ×400).

**Figure 10:**
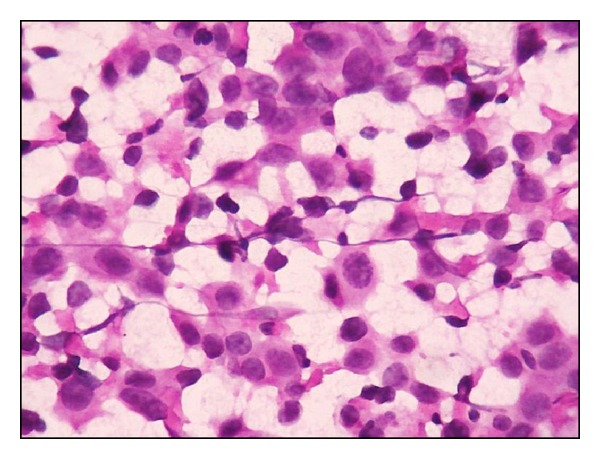
Howell's grade II. Tubule formation, moderate nuclear pleomorphism (H&E ×400).

**Figure 11:**
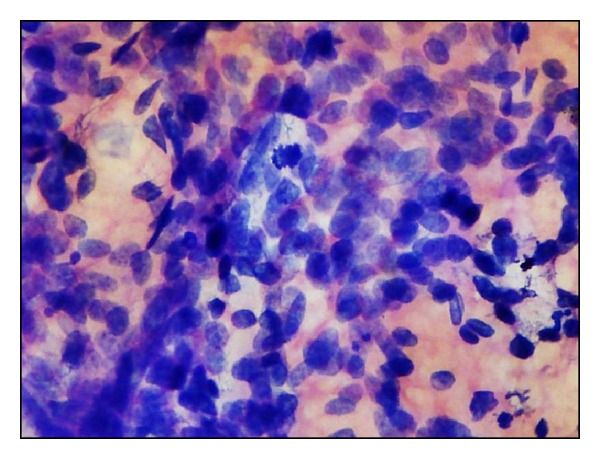
Howell's grading. Mitotic figure (H&E ×400).

Mouriquand and Pasquier [12] (Figure 9) gave a score of 0–3 to cellular and nuclear features, chromatin and mitosis and graded as I if total score was <5, II if in the range of 6–9, and III if >10. Howell's system [13] (Figures 10 and 11) is similar to the Scarff-Bloom-Richardson histological grading with modification to the mitotic count as score 1 for 0-1/10 high power fields, 2 for 2–4/10 hpf, and 3 for >5/10 hpf. Grades were allotted as I, II, and III for scores in the range of 3–5, 6-7, and 8-9, respectively.

**Figure 12:**
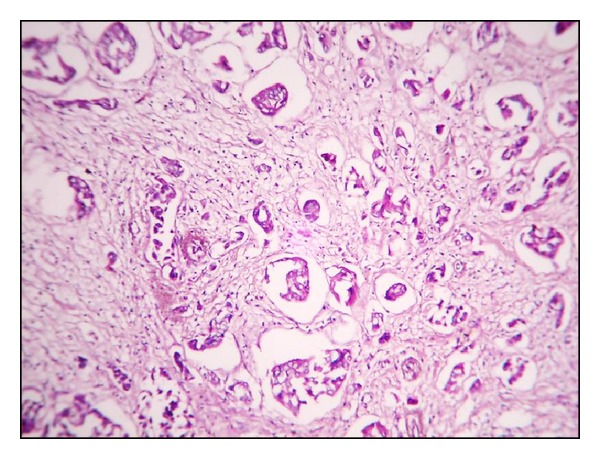
Histological grade I by SBR (H&E ×40).

**Figure 13:**
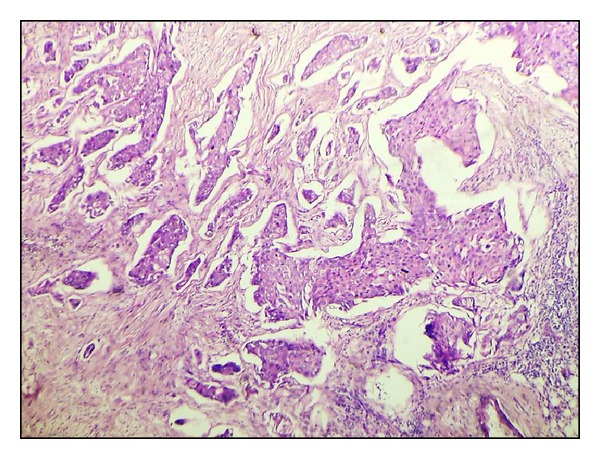
Histological grade II by SBR (H&E ×40).

**Figure 14:**
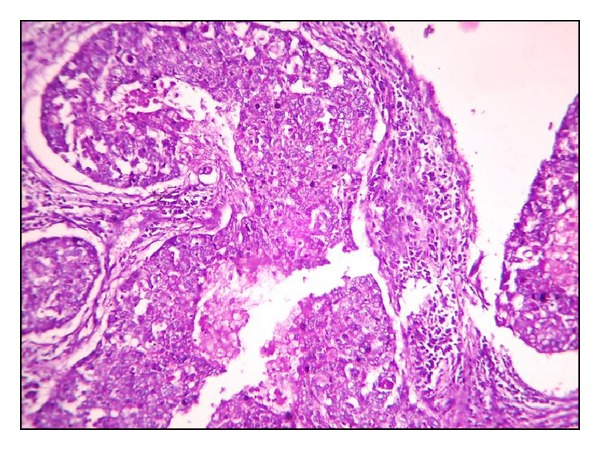
Histological grade III by SBR (H&E ×40).

The corresponding H&E stained sections of the formalin-fixed, paraffin embedded blocks of post-operative mastectomy specimens were studied and the histological grading done by Nottingham modification of Scarff-Bloom-Richardson's method [14] (Figures 12, 13, and 14). Mitotic figures were counted and scored using an Olympus CH20i microscope with high power field diameter of 0.45 mm.

The results were tabulated and statistical analyses were done with the IBM SPSS statistics software, version 20. The association between the grading systems was assessed by Chi-Square test. *P* value < 0.001 was considered as statistically significant. Correlations were judged by Spearman's correlation coefficient (*ρ*). Agreement/concordance was assessed by Kappa (*κ*) measurement of agreement.

## 3. Results 

FNAC and histopathology of 72 cases of invasive ductal carcinoma of breast were studied. The age distribution of cases was in the range of 34–79 years. Majority of the cases were grade II (44 out of 72 cases). The distribution of cases according to the cytological grading systems and histological grading is listed in Table 1.

The association between each of the cytological grading systems and the histological grading was found to be highly significant with a *P* value of <0.001, as measured by the Chi-square test. The correlation and concordance analyses between the cytological grading and histological grading were as in Table 2.

All the 7 cytological grading systems correlated well with the histological grading, as calculated by Spearman's correlation coefficient (*ρ*). Robinson's system showed the highest concordance (56 out of 72 cases, 77.7%) and agreement (*κ* value 0.61, substantial agreement), with the histological grading, closely followed by fisher's system (55 out of 72 cases, 76.3% and *κ* = 0.526, moderate agreement), whereas Taniguchi's system showed the least concordance (48 out of 72 cases, 66.6%) but fair agreement with Kappa value of 0.401.

The interobserver agreement was analysed by Kappa measurement of agreement and the result observed was recorded in Table 3.

All the grading systems showed moderate to substantial inter-observer agreement. Fisher's system showed the highest concordance (61 out of 72 cases, 84.7%, *κ* = 0.616) of grading between the two pathologists, followed by Robinson (83.3%), Dabbs and Silverman (80.5%), and Taniguchi (80.5%) systems. Interobserver agreement for histological grading was almost perfect (*κ* = 0.921).

## 4. Discussion 

Fine-needle aspiration cytology plays a major role in the primary diagnosis of breast carcinoma. Cytological grading of the smears can provide valuable prognostic information and aid in planning the management options. Several grading systems have been proposed by various authors, but none have been implemented successfully in routine practice. This study was done with the aim of finding a reliable cytological grading system which is not only simple and effective, but also better agreed among the reporting pathologists.

Various studies have highlighted the benefits of Robinson's system, but fewer studies have considered other three-tier systems of cytological grading. In this study, we have compared 7 three-tier cytological grading systems with the histological grading and also the interobserver agreement. To increase the credibility of the assessment, interobserver agreement for histological grading was also done, which came to be almost perfect. Our results proved to be in favour of Robinson's system; however, Fisher's system has also shown to be a good alternative.

Different studies in the past have shown varying concordance between cytological and histological grading. Bhargava et al. [15] 77.78%, Zoppi et al. [16] 70.37%, Saha et al. [17] 70.18% and in the present study 76.3% of concordance, between Fisher's system and histological grading.

Comparison studies between Robinson's grading and histological grading showed agreement of 57% by Robinson et al. [7], 80.76% by Das et al. [18], 88.89% by Bhargava et al. [15], 65% by Chhabra et al. [19], 83% by Meena et al. [3], 88% by Khan et al. [20], 81% by Sinha et al. [21], 64% by Lingegowda et al. [22], 77.19% by Saha et al. [17], and 77.7% in the present study. The advantage of this system is that the effect of individual variation in the evaluation of a single component of the system is reduced by analysis of the other components, as stressed by Dalton et al. [23].

We observed concordance of 72.2% for Dabbs and Silverman grading with histological grading, whereas Dabbs and Silverman [9] observed 87%. Khan et al. [10] found concordance of 97.14% for the grading system proposed by them, but in the present study, we observed 72.2%. Taniguchi's system yielded concordance of 66.6% in our study, whereas Taniguchi et al. [11] observed 44.4% only.

Das et al. [18] compared Robinson's and Mouriquand systems with the histological grading and observed similar results (71.2% concordance) but considered Robinson's method as a better choice due to its simplicity, specificity, and better reproducibility. Our study showed a concordance rate of 68% for Mouriquand's system and 69.4% for Howell's system, whereas it was 57.1% by Howell et al. [13], 50% by Bhargava et al. [15], 82% by Lingegowda et al. [22], and 63.16% by Saha et al. [17] for Howell's grading, which was a modification of the Nottingham's Scarff-Bloom-Richardson grading.

Very few studies on interobserver agreement are available. Howell et al. [13] found inter-observer agreement of 74.3% for histological grading and 65.7% for cytological grading, Lingegowda et al. [22] found 98% interobserver agreement for Robinson's system compared to 92% for Howell's system. The study by Saha et al. [17] showed an interobserver agreement of 78.95% for Fisher's system, 84.21% for Mouriquand's, 77.19% for Robinson's, 85.96% for Howell's, 80.70% for Khan's, and 80.70% for Taniguchi's systems. In comparison, our study had 84.7% for Fisher's, 83.3% for Robinson's, 80.5% for Dabbs', 79.1% for Khan's, 80.5% for Taniguchi's, 76.3% for Mouriquand's, and 75% for Howell's system.

The major drawbacks observed were using cell pattern as criteria. As it is also an important criterion for diagnosis of malignancy, once the diagnosis of malignancy is established, the smear will show a score of 2 for cellular pattern. But various authors had determined by regression analysis, extent of cell dissociation as the most influential factor for scoring along with the appearance of nucleoli. Subtle degrees of nuclear pleomorphism and loss of cell-to-cell cohesion were difficult to score, resulting in minor discrepancies. Also, we felt difficulty in detecting mitosis in the aspiration smears, as observed by Howell et al. [13] too. This may be due to the fact that, in cytological smears, the material aspirated is very less compared to that available in histological sections.

Other factors which contributed to difficulty in grading included poor quality slides with fixation artifacts and tumours with necrosis or severe inflammation.

## 5. Conclusion 

Cytological grading of breast carcinoma is feasible and provides valuable prognostic information. We propose that it be included in FNAC reports of breast malignancies. In the present study, all the seven cytological grading systems correlated positively with the histological grading. However, Robinson's method showed a better correlation and substantial Kappa value of agreement with the histological grading in comparison to the other methods. This is because of the multifactorial nature of the system. According to our study, Robinson's method of cytological grading in fine needle aspiration smears of breast carcinoma is simpler, objective and easily reproducible, hence being preferable for routine use.

## Figures and Tables

**Table 1:** Distribution of cases according to the cytological and histological grading.

Grade	Fisher's system	Robinson's system	Dabbs' system	Khan's system	Taniguchi's system	Mouriquand's system	Howell's system	Histological grading
I	7	12	9	6	19	17	15	11
II	55	40	50	38	44	45	46	44
III	10	20	13	28	9	10	11	17
Total	**72**	**72**	**72**	**72**	**72**	**72**	**72**	**72**

**Table 2:** Correlation and concordance analyses between the cytological grading systems and the histological grading.

	Fisher's system	Robinson's system	Dabbs' system	Khan's system	Taniguchi's system	Mouriquand's system	Howell's system
Correlation (Spearman *ρ*)	0.654	0.738	0.604	0.696	0.615	0.613	0.614
Concordance	55/72	56/72	52/72	52/72	48/72	49/72	50/72
	76.3%	77.7%	72.2%	72.2%	66.6%	68%	69.4%
Agreement (kappa *κ* )	0.526	0.61	0.459	0.515	0.401	0.418	0.436
	(moderate)	(substantial)	(moderate)	(moderate)	(fair)	(moderate)	(moderate)

**Table 3:** Analysis of inter-observer agreement for cytological grading systems.

	HP	Fisher's system	Robinson system	Dabbs' system	Khan's system	Taniguchi's system	Mouriquand's system	Howell's system
Inter-observer agreement	69/72	61/72	60/72	58/72	57/72	58/72	55/72	54/72
	95.8%	84.7%	83.3%	80.5%	79.1%	80.5%	76.3%	75%
Kappa value	0.921	0.616	0.708	0.56	0.615	0.618	0.561	0.499
	Almost perfect	substantial	substantial	moderate	substantial	substantial	moderate	moderate
